# A rare case of Alport syndrome, atypical hemolytic uremic syndrome and Pauci-immune crescentic glomerulonephritis

**DOI:** 10.1186/s12882-018-1170-4

**Published:** 2018-12-12

**Authors:** Jianling Tao, Jonathan Lieberman, Richard A. Lafayette, Neeraja Kambham

**Affiliations:** 1 Department of Medicine, Division of Nephrology, Stanford University, Stanford, USA; 2 Department of Nephrology, Kaiser Permanente, San Francisco, CA USA; 3 Department of Pathology, Stanford University, Stanford, CA USA

**Keywords:** Atypical hemolytic uremic syndrome, Pauci-immune glomerulonephritis, Alternative complement pathway, Thrombotic microangiopathy

## Abstract

**Background:**

Renal thrombotic microangiopathy (TMA) is occasionally seen in biopsies with pauci-immune necrotizing crescentic glomerulonephritis (PCGN). Recent study indicated that the complement activation is more prominent in the ANCA-negative glomerulonephritis.

**Case presentation:**

We report a case of concurrent TMA and PCGN without ANCA positivity. Interestingly, our patient also had biopsy features supportive of Alport syndrome (AS). Genetic studies identified variants and polymorphisms in alternative complement pathway genes that confer substantial risk of developing atypical hemolytic uremic syndrome (aHUS).

**Conclusions:**

Abnormal activation in complement pathway may represent a common pathogenic link between these three distinct entities.

## Background

Over the last several years, the causative role of the alternative complement pathway in atypical hemolytic uremic syndrome (aHUS) has been confirmed. Anti-complement therapy has been shown to be beneficial in aHUS. Pauci-immune crescentic glomerulonephritis (PCGN) lacks immune complex deposition and has not traditionally been associated with complement abnormalities. We present a patient with biopsy evidence of thrombotic microangiopathy (TMA), Alport syndrome (AS) and glomerular crescents and discuss potential pathogenic link.

## Case report

### Clinical Presentation

A 26-year-old woman presented with progressive shortness of breath. Her past medical history was notable for asthma diagnosed at age 3, hearing loss at age 23 (that was not fully worked up) and hypertension at age 24. Ten months prior to admission, her blood pressure was 143/91 mmHg, and urine dipstick detected large protein and blood, but she was lost to follow up. The family history was significant for asthma and hypertension in her mother, type II diabetes mellitus in her father and asthma in her brother; her sister was healthy.

On admission, she was afebrile, blood pressure was 191/125 mmHg and the physical examination was otherwise unremarkable; fundoscopy was not performed. Urine analysis detected 25–50 non-dysmorphic RBCs/HPF, 5–10 WBCs/HPF,100 mg/dl protein, positive leukocyte esterase, and numerous “muddy brown casts”. Relevant laboratory results were white blood cell count 7.2× 10^9^/L, hemoglobin of 8.9 g/dL, platelet count 73,000/μL, BUN 71 mg/dL, and serum creatinine 10.11 mg/dL. Other studies include AST 34 u/L, ALT 36 u/L, haptoglobin 10 mg/dl (low), and LDH 2331 u/L (very high). Occasional schistocytes were seen on peripheral smear and ADAMTS13 was normal. Serological studies were normal including C3 (138), C4 (37.2), ANA (neg), anti-MPO (< 0.2), anti-PR3 (< 0.2), anti-GBM antibodies (< 0.1), hepatitis B, hepatitis C, HIV, and serum and urine immunoelectrophoresis. Chest x-ray showed pulmonary congestion and kidneys were echogenic on ultrasound. The patient was started on hemodialysis and a kidney biopsy was performed.

### Kidney Biopsy #1

**Fig. 1:**
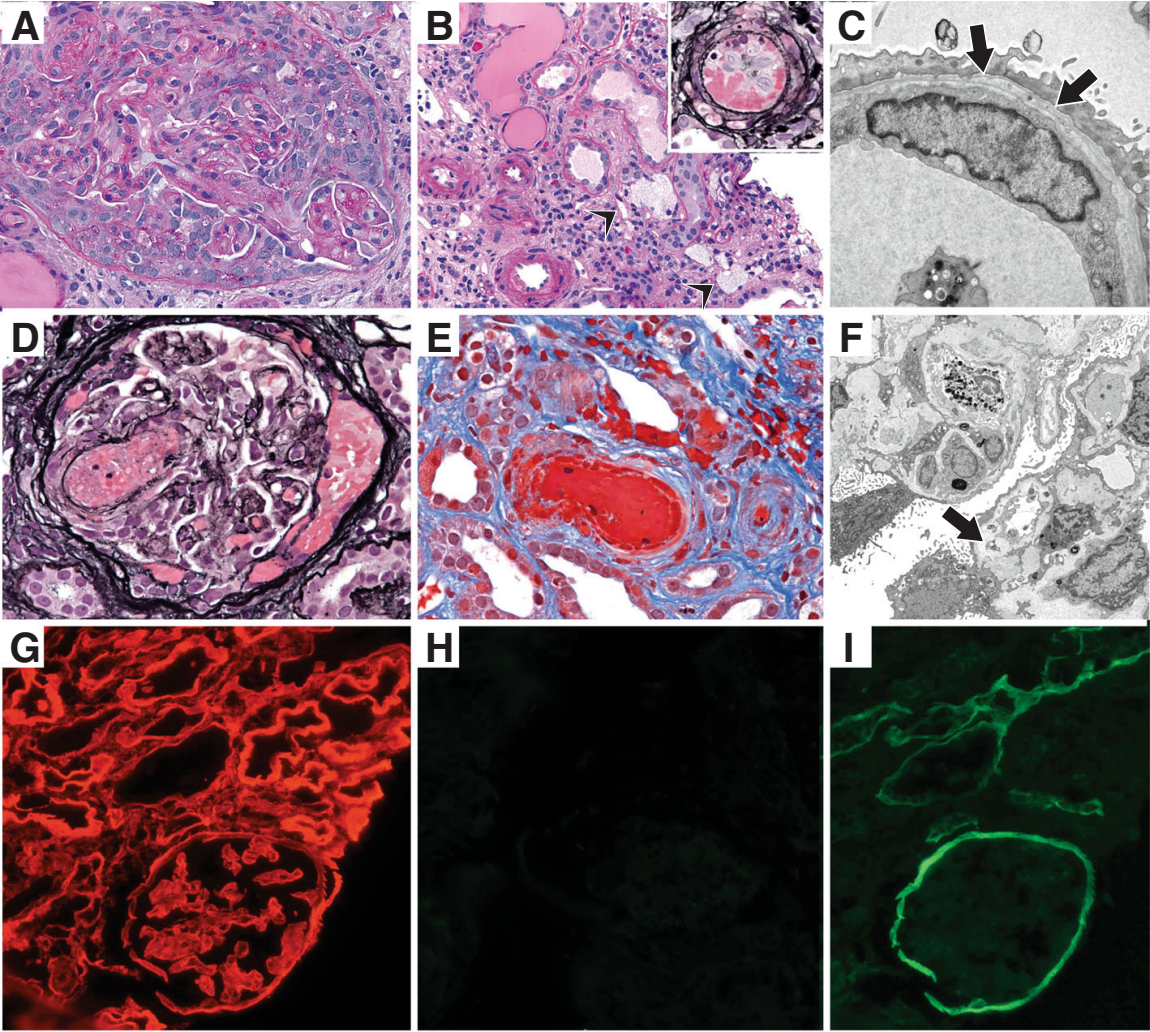
Renal Biopsy Findings: **a-c**: Biopsy #1. **a**: A circumferential cellular crescent is seen (PAS, × 400) **b**: There is prominent tubular atrophy and interstitial fibrosis along with interstitial foam cells (arrowheads) (H&E, × 300). Most arterioles are mildly thickened, but an arteriole shows intraluminal fibrin with karyorrhectic debris (inset, JMS) **c**: Lamina densa lamellation (arrows) is seen along the glomerular basement membrane on electron microscopy (× 8000). **d-i**: Biopsy #2. **d**: Glomerulus with fibrin thrombus and fibrinoid necrosis near the vascular pole (JMS × 400) **e**: Arteriole with fibrin thrombus, karyorrhectic debris and extravasated red blood cells. No vasculitis is seen (trichrome × 400). **f**: Ischemic retraction and thickening of the glomerular basement membranes on electron microscopy. Mesangiolysis and expansion of the subendothelial space is seen (arrow) (× 3000). **g**: Tissue antigen preservation documented with type IV collagen α2 staining **h**: Complete absence of α3 staining in the glomerular and tubular basement membranes **i**: Absent glomerular basement membrane staining with type IV collagen α5. But linear staining along the Bowman’s capsule and distal tubular basement membranes is preserved

The light microscopy sample had only 2 glomeruli and they had circumferential cellular crescents (Fig. 1a) and compressed capillary loops. Prominent tubular atrophy and interstitial fibrosis was seen with sparse interstitial inflammation and a few interstitial foam cells. The interlobular arteries and arterioles were mostly well preserved, but one arteriole had endothelial swelling, karryorrhexis and fibrin thrombus (Fig. 1b). No vasculitis was identified. Immunofluorescence revealed one glomerulus (also with crescent) and segmental granular C3 (1+; scale 0–4). No staining was seen with IgG, IgA, IgM, C1q, κ and λ. Three glomeruli, again with crescents, were available for ultrastructural examination. In addition to the extravasated fibrin and basement membrane rupture, focal endothelial cell damage was apparent with expansion of the subendothelial space by electron lucent material. Several capillary loops had basement membrane irregularities and multi-lamination of lamina densa (Fig. 1c). No electron dense deposits were identified and podocytes overlying these compressed capillary loops displayed prominent foot process effacement.

The biopsy findings were most compatible with crescentic glomerulonephritis without extrarenal manifestations. Occasional arterioles had histological features of TMA. The glomerular basement membrane changes raised concern for Alport syndrome (AS), but due to the paucity of tissue sample, type IV collagen staining could not be performed. Given the suboptimal sample and concern for systemic TMA and AS, a second biopsy was performed 3 weeks after admission.

### Kidney Biopsy #2

Eighteen glomeruli were sampled for light microscopy and three were globally sclerosed. Cellular/fibrocellular crescents were seen in 8 glomeruli with one fibrous crescent. Fibrin thrombi were identified in two glomeruli (Fig. 1d). Several other glomeruli had ischemic changes and the tubular atrophy and interstitial fibrosis involved over 80% of the cortex. Clusters of interstitial foam cells were seen throughout the cortex. Several cross sections of interlobular arteries and arterioles had intimal edema, fibrinoid necrosis, and concentric thickening of the wall; no vasculitis was identified (Fig. 1e). Immunofluorescence was negative. On ultrastructural examination of 3 ischemic glomeruli, extensive endothelial cell injury was seen with subendothelial electron lucent material; prominent mesangiolysis was also present (Fig. 1f). Lamina densa lamellation was again seen. Additional immunofluorescence was performed for type IV collagen α2, α3 and α5 chains (Fig. 1g-i). Alpha2 staining was normal, indicative of good antigen preservation. However, α3 was completely absent in both glomeruli and tubules, and α5 lacked staining along the glomerular basement membranes but had intact staining along Bowman’s capsules and distal tubular basement membranes.

### Immunohistochemical Studies

**Fig. 2:**
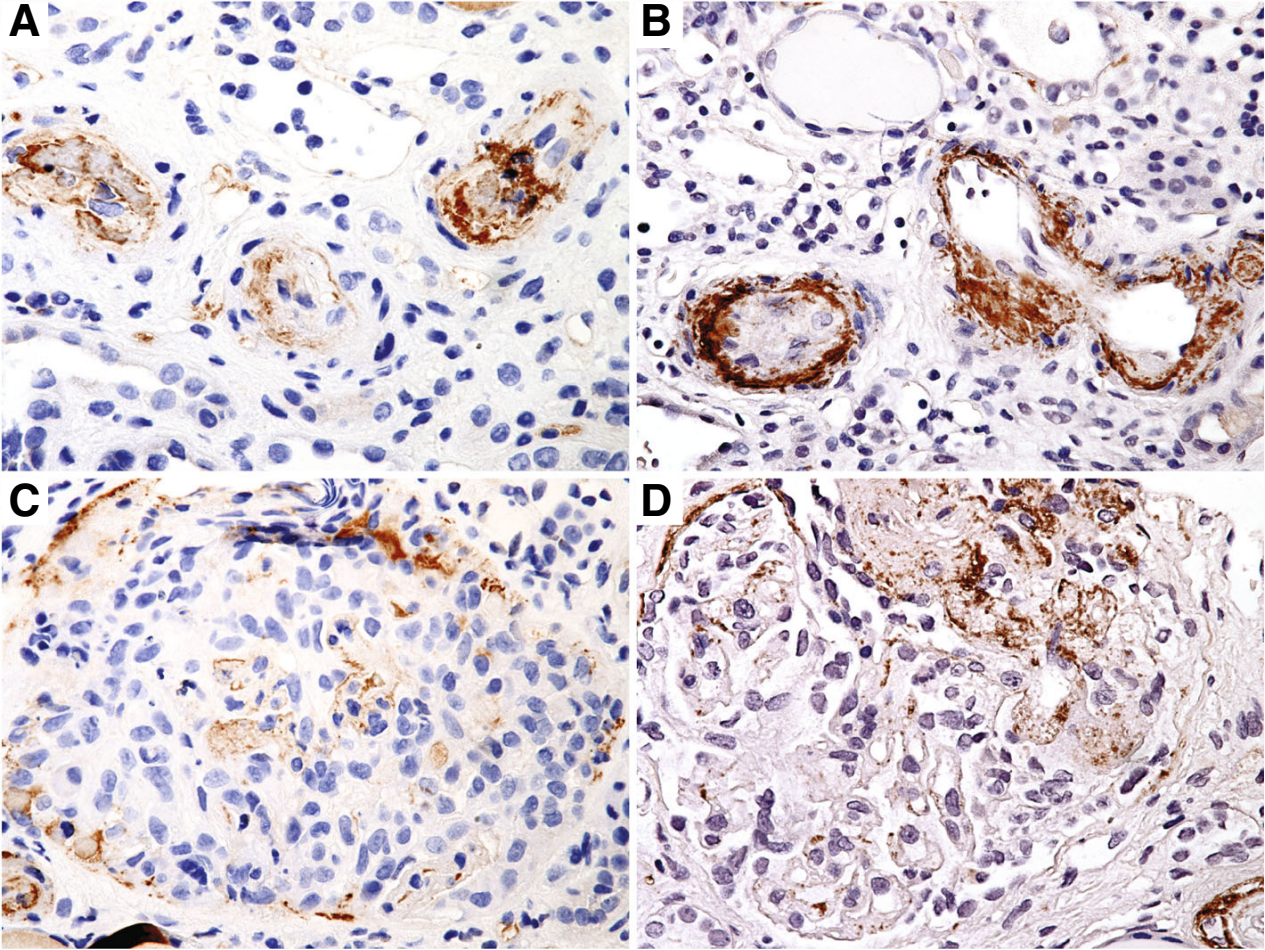
Immunohistochemical evidence of complement pathway activation. **a**: Arteriolar C4d staining within intimal and adjacent media in vessels affected by acute thrombotic microangiopathy (biopsy #1) (× 400) **b**: Arteriolar C5b-9 (membrane attack complex) staining in affected vessels (biopsy #1) (× 400) **c**: Weak glomerular staining with C4d in a crescent (× 400) **d**: Glomerular C5b-9 staining predominantly within a cellular crescent (biopsy #2) (× 400)

Both kidney biopsies were stained for C4d (rabbit polyclonal, cat. no. 04-B1-RC4D, Biomedica, Austria, 1/20 dilution) and C5b-9 (mouse monoclonal, cat.no. M0777, DAKO, Denmark, 1/10 dilution). Intimal and medial staining was seen with both C4d and C5b-9 in arterioles affected by thrombotic microangiopathy. Glomerular staining was seen in crescents and sclerosed segments, albeit more strongly with C5b-9 (Fig. 2). In addition, C5b-9 staining was seen in patchy peritubular capillaries and interstitium.

### Diagnosis and follow-up

Both kidney biopsies demonstrate crescentic glomerulonephritis, with increased chronicity on the second biopsy. In addition, there was acute and subacute TMA, better visualized on second biopsy. A sequencing of 12 aHUS-related genes was performed using a database of over 400 aHUS-associated mutations, disease-associated polymorphisms, benign polymorphisms and other known variants of undetermined significance (Machoan Diagnostics, Oakland, CA). Our patient was negative for the large *CFHR1-CFHR3* homozygous deletion associated with CFH auto-antibodies. However, she had a heterozygous missense variant (c.1246 A > C, p.lle416Leu) in exon 11 of *CFI,* reported in aHUS [1, 2]. A heterozygous missense variant (c.136C > T, p. Pro46Ser) in exon 2 of *CFHR5*, reported in dense deposit disease was also noted [3]. A heterozygous polymorphism (IVS9–78 G > A) within an intron in *MCP/CD46* was present and there were also 5 SNPs near and within *MCP/CD46* that comprised a haplotype, also associated with increased risk for aHUS [4].

The clinical, laboratory and genetic findings indicate a diagnosis of aHUS coexistent with glomerular crescents seen on biopsy. The interstitial foam cells on light microscopy (in the absence of chronic nephrotic proteinuria), basement membrane abnormalities on electron microscopy and abnormal immunofluorescence for α3 and α5 chains of type IV collagen support a diagnosis of AS, that likely contributed to the underlying chronic kidney disease. The immunofluorescence pattern of α3 and α5 staining suggests a α3 gene mutation with autosomal recessive inheritance. A genetic analysis for AS was suggested.

The patient was treated on admission with pulse methylprednisolone followed by oral prednisone. Daily plasmapheresis was initiated, but was discontinued after a few days once the ADAMTS13 levels were found to be normal. Cytoxan was administrated after the first biopsy results. Eculizumab therapy was initiated after the second biopsy and she continued to receive it every 2 weeks at her 8-month follow up. Her kidney function remains poor requiring maintenance hemodialysis.

## Discussion and conclusions

Atypical hemolytic uremic syndrome (aHUS) is relatively rare, and studies over the last decade have vastly improved our understanding of the disease [5, 6]. In approximately 60% of cases, aHUS is linked to mutations in complement genes encoding both regulatory and activation proteins involved in the complement pathway. Disease results from either a loss-of-function mutation in a regulatory gene (eg. *CFH*, *CFI*, or *CD46*) or a gain-of-function mutation in an effector gene (eg. *CFB* or *C3*), resulting in alternative complement pathway dysregulation on cell surfaces. *CFH* mutation-associated aHUS, the most common form, comprises 20–30% of inherited disease [7]. In a subset of patients, similar to ours, combinations of gene abnormalities result in aHUS [5]. The disease penetrance is variable and several environmental triggers such as infections, pregnancy, respiratory illness and drugs likely precipitate overt aHUS. The precipitating factor in our patient is unclear. Only a third of aHUS patients have low serum C3 levels, usually in association with complement gene mutations. The biopsy findings of aHUS are those of TMA involving glomeruli and/or small arteries and arterioles.

The prevalence of alternative complement pathway dysregulation in disease appears to be greater than previously appreciated. Timmermans et al [8] examined complement abnormalities in nine patients with malignant hypertension-associated TMA, and found mutations in *C3, CFI, CD46,* or *CFH* genes in six. Complement activation in these patients was evidenced by elevated serum soluble C5b-9 levels and renal deposits of C3c and C5b-9. Co-localization of C4d deposits in vasculature and glomerular capillary walls indicates activation of classical and/or lectin pathway as well. Our patient did show systemic evidence of TMA (akin to malignant hypertension) with circulating schistocytes and elevated LDH levels. Chua et al [9] evaluated for C4d and C5b-9 deposits in 42 renal biopsies (both native and transplant) with TMA related to heterogeneous underlying diseases. C4d and C5b-9 deposits were quite common, although the pattern of staining was variable based on etiology. Our patient had deposits of both C4d and C5b-9, providing immunohistochemical support for complement pathway activation. Continuous alternative complement pathway abnormalities in aHUS patients result in endothelial dysfunction and formation of microvascular thrombi. Eculizumab is a humanized monoclonal IgG antibody that binds to complement protein C5, preventing cleavage into C5a and C5b. Blocking the formation of C5b interrupts this complement cascade and membrane attack complex (C5b-9)-mediated injury. Several studies have shown the safety and efficacy of eculizumab therapy in patients with aHUS [10].

Alport syndrome (AS), which usually manifests as a triad of hematuria, senisorineural hearing loss and ocular symptoms, is caused by the disruption of type IV collagen, a key component of the glomerular basement membrane. Specifically, mutations in the *COL4A5*, *COL4A3* or *COL4A4* genes, which encode the α3, α4, and α5 chains of collagen type IV respectively, cause the disease [11]. Over 500 mutations have been described, which result in disease with either X-linked (mutations in *COL4A5*; 85% of patients) or autosomal recessive inheritance (mutations in *COL4A3* and *COL4A4*; 15% of patients) [12]. An autosomal dominant inheritance pattern has been reported in a minority of patients [12, 13].

A diagnosis of AS is generally determined by the family history, presence of visual and/or hearing dysfunction, and electron microscopy findings on kidney biopsy. The glomerular basement membrane changes are typically characterized by diffuse thickening and splitting of the lamina densa. Immunofluorescence in our patient revealed total lack of α3 and loss of α5 staining in glomerular basement membranes but preserved α5 staining in Bowman capsule and distal tubular basement membranes. Mutations in the *COL4A3* (or *COL4A4)* gene results in the defective triple helix with demonstrable α5 protein loss only in those basement membranes (glomerulus) in which all three proteins are associated. [14] Based on these findings, we suspect that our patient has autosomal recessive AS.

Crescents are rarely reported in aHUS [15], but tend to be small and barely meet the criterion for a crescent [16]. It is also possible that the crescents in our patient are related to AS [17]. In reviewing 665 human Alport nephropathy biopsies, Ryu et al. [18] found glomerular crescents in 0.4% of biopsies, suggesting an extremely rare occurrence. In each of those biopsies, only 3–11% of glomeruli sampled had crescents. It has been proposed that plasma leakage due to glomerular vascular injury and glomerular basement membrane breaks might trigger parietal epithelial cell proliferation and crescent formation. Based on studies in Col4A3 –deficient mice, crescent formation occurs more frequently in late stage Alport glomerulopathy [18].

The presence of extensive crescents seen in our patient also suggests co-existent ANCA negative (antineutrophil cytoplasmic antibody anti-PR3 and anti-MPO) pauci-immune crescentic glomerulonephritis (PCGN). Review of the literature demonstrates a growing appreciation for the role of complement in PCGN [19, 20]. Complement plays a key role in murine ANCA-associated PCGN induced by injection of anti-MPO antibodies [21]. The alternative complement pathway has been shown to be critical in the pathogenesis as mice deficient in factor B or C3 fail to develop PCGN [21]. Anaphylatoxin C5a and the neutrophil C5a receptor mediate an amplification loop for ANCA-induced neutrophil activation that triggers the complement cascade in the serum. C5a, a potent chemoattractant for neutrophils, monocytes and macrophages, also increases the membrane expression of ANCA on neutrophils, leading to reactive oxygen species generation and necrotizing vasculitis [22].

Despite the general impression, a high percentage of patients with ANCA-associated PCGN have immunofluorescence and ultrastructural evidence of immune deposits. These immunoglobulin and complement deposits are typically of low intensity [23]. Recently, Chen et al. examined 112 biopsies from patients from ANCA-associated PCGN [24]. In one third of the patients, glomerular C3c deposits were detected and this feature was associated with greater proteinuria and worse renal function at presentation. Other components of alternative complement pathway activation such as factor Bb, C3d and properdin deposits were also demonstrable in the kidney biopsies with ANCA-associated vasculitis [25–27] .The presence of C3d and properdin deposits correlated with more severe crescentic disease [27]. Other investigators have sought evidence of alternative pathway activation and complement consumption in serum and urine. Low serum C3 level at diagnosis of renal ANCA vasculitis is associated with lower renal function at presentation and worse renal and patient survivals [28]. Circulating serum factor Bb is elevated in active ANCA vasculitis and may in fact be a biomarker for disease activity [29]. Alternative complement pathway activation products Bb, C3a, and C5a and common terminal pathway product soluble C5b-9 were all found to be significantly elevated in the urine of patients with active compared to remission stage of ANCA PCGN [25]. There may also be activation of classic and lectin complement pathways, but the overall evidence suggests that they do not play a critical pathogenic role [25, 27, 30].

Given the involvement of alternative complement pathway in both ANCA disease and aHUS, it seems quite plausible that these diseases occur concurrently, as has been rarely reported [31]. Manenti et al. studied 46 patients with ANCA vasculitis, and eight of 30 patients who underwent biopsy (27%) had histologic signs of TMA [32]. This subgroup with histological evidence of TMA had a dramatically worse death-censored renal survival than patients without TMA. Interestingly, serum C3 levels were low in more than a third of these patients with ANCA vasculitis. Identifying a pathogenic role for complement in PCGN disease has significant therapeutic implications. Initiation of anti-complement therapy (eculizumab, anti-C5) immediately after a diagnosis of aHUS is associated with better renal outcome [33]. There can be a potential beneficial role of anti-complement therapy in PCGN disease as well [34, 35]. Recently, Sathe et al. [36] reported a pediatric patient with biopsy proven ANCA disease who developed aHUS 6 months later, as evidenced by low serum C3 and circulating anti-factor H antibodies. No genetic studies were performed. Complete renal recovery was achieved following treatment with intravenous rituximab, steroids, cyclophosphamide, and plasmapheresis.

Overall, it seems that TMA in ANCA disease may be under recognized both clinically and on biopsy. The identification of at risk genetic variants and polymorphisms in complement genes in our patient with both PCGN and TMA lends further support for shared pathogenic pathway in these two histologically distinct entities. Ours is a rather unusual case with a third diagnosis of AS. Glomerular crescents can rarely be seen in AS, and there are rare reports of TMA in an AS patient [37, 38]). A 16 year old patient presented with nephrotic syndrome and the biopsy revealed chronic TMA and a diagnosis of X-linked AS was subsequently made based on whole-exome sequencing. So, while a PCGN superimposed on TMA is a distinct possibility, crescent formation may have been triggered or accentuated by both glomerular injury caused by aHUS and the compromised basement membrane integrity due to AS.

In conclusion, we report a case of AS patient with concurrent aHUS and glomerular crescents. Genetic analysis revealed at risk variants and polymorphisms in alternative complement pathway genes. We hypothesize that this genetic predisposition in this unfortunate patient led to precipitation of aHUS and potentially PCGN in the context of an unidentified environmental trigger.

## Abbreviations

aHUS: Atypical hemolytic uremic syndrome
AS: Alport syndrome
PCGN: Pauci-immune necrotizing crescentic glomerulonephritis
TMA: Renal thrombotic microangiopathy
